# Factors Associated with Mothers’ Care of Their Newborns in Saudi Arabia

**DOI:** 10.5334/aogh.2524

**Published:** 2019-07-11

**Authors:** Ibrahim M. Gosadi, Hadi H. Daghreeri, Jnadi M. Madkhali, Alanoud I. Mokhasha, Zainab A. Athwani, Mohssen H. Ageeli, Ahmed A. Bahri, Ghadah M. Gosadi

**Affiliations:** 1 Department of Family and Community Medicine, Faculty of Medicine, Jazan University, Jazan, SA; 2 Department of Paediatrics, King Fahad Central Hospital, Jazan, SA

## Abstract

**Background:**

Infant mortality rates are highest in the southern regions of Saudi Arabia, compared to other regions in the kingdom.

**Objective:**

To measure demographic factors associated with mothers’ levels of knowledge and practice of care for their newborns in Jazan region, south of Saudi Arabia.

**Methods:**

This is a cross-sectional study conducted between November and December 2018 in Jazan region, Saudi Arabia, on the northern borders of Yemen. A questionnaire was utilised to measure mothers’ level of knowledge and practice of newborn care. Data was collected via interviews, and a scoring system was developed to classify knowledge level and practice adequacy. Logistic regression was used to assess the presence of statistically significant associations between demographic factors and level of knowledge and practice adequacy.

**Findings:**

A total of 450 mothers participated in the current investigation. A majority of participating mothers were able to give correct answers, where the mean level of knowledge was 11.85/16 [SD: 2.6]. Additionally, the mean score for practice adequacy was 7.11/10 [SD: 1.45]. However, 122 mothers (27%) reported using alternative treatments to treat their newborns instead of seeking professional health care from available health services. Additionally, 42 mothers (9.3%) reported not attending any antenatal visits during their pregnancy. Factors which were found to be statistically associated with knowledge were education level, employment status, and adherence to antenatal visits during pregnancy (p < 0.05). Age and employment status appeared to be associated with practice where older and employed mothers had higher odds of competency (p < 0.05).

**Conclusions:**

The proportions of correct answers measuring knowledge and practice adequacy concerning newborn care varied between 40% to 93%. Knowledge and practice appeared to be associated with demographic factors, such as level of education, age, and attending antenatal care visits.

## Introduction

The neonatal period refers to the period less than 28 days after birth [1]. The Global Health Observatory reported that 2.6 million neonatal deaths occurred during 2016, which represents 46% of all deaths under 5. Additionally, the majority of deaths among newborns occur during the first day and week of birth [2].

Those who die in the infancy period experience conditions and diseases that can be managed with adequate and rapid quality care after delivery [3]. Possible aetiologies of such rising neonatal deaths include prematurity, birth-related complications, neonatal sepsis, and, in older children, pneumonia and diarrhoea [4]. Moreover, lack of exclusive breastfeeding in the first five months of life increases the risk of death from diarrhoea sevenfold and from pneumonia fivefold [5].

According to the World Health Organization (WHO), neonatal mortality rates in Saudi Arabia dropped from 22 per 1,000 live births in 1990 to 7 in 2016. Additionally, in a national survey conducted in Saudi Arabia involving 10,931 ever married women, it was concluded there had been a reduction in the infant mortality rate from 29 to 20 deaths per 1,000 live births between 1994 and 2004 [6].

Variations in infant mortality between different regions in Saudi Arabia are apparent. According to Al-Mazrou et al. infant death rates were highest in the southern regions of Saudi Arabia [6]. Additionally, the Saudi General Authority for Statistics reported that neonatal mortality rates were highest in Najran region (5.91) and third highest in Jazan region (3.85); both these regions are in the south of Saudi Arabia [7].

Variations in neonatal mortality rates in different regions of Saudi Arabia can be explained by variations in the availability and quality of care provided during and after delivery. Furthermore, variations in the level of knowledge of mothers concerning neonatal care may induce variations in practice and appropriate utilisation of available health services.

Appropriate knowledge about newborn care is crucial to enhance practice and reduce the risk of neonatal problems and death. According to the Saudi Ministry of Health Statistics Book of 2017, Jazan region was ranked fourth in the number of total deliveries in hospitals of the ministry [8]. The high number of births and high neonatal mortality rate in Jazan region mandates investigating the factors associated with mothers’ care of newborns in Jazan. The level of knowledge among mothers and their practice concerning newborn care in Jazan is currently unknown. This study was conducted to help fill that gap.

## Methods

### Study Settings and Participants

This is a cross-sectional study conducted in Jazan region of Saudi Arabia between November and December of 2018. The study was performed in 11 Primary Health care Centres (PHCs) in Jazan region, including the 5 largest cities: Gizan, Sabia, Abu-Arish, Al-Ahad, and Samta. Ethical approval to conduct the study was granted by Jazan Hospital Institutional Review Board, Ministry of Health (approval number 1833 dated 28/06/2018). Informed consent to participate was obtained from mothers before their recruitment.

The target population of this investigation was Saudi mothers attending PHCs whose youngest child was younger than 5 years. Mothers whose youngest child was older than 5 years were not targeted to reduce the magnitude of recall bias concerning newborn practice. Several PHCs were targeted in different sectors of Jazan region to enhance the generalisability of the findings.

The sample size for this study was calculated based on a mother’s knowledge regarding child healthcare. Al-Ayed conducted a study in Riyadh, Saudi Arabia, to measure mothers’ knowledge regarding child health [9]. In Al-Ayed’s study, several questions were asked to assess the mother’s knowledge, where the lowest proportion of mothers able to give the correct answer to a question was 16%. This prevalence was taken into consideration for calculating the sample size for the current study to increase the study’s power to detect true prevalence of knowledge. A sample of 460 subjects was estimated using the formula for a prevalence assessment, n = ([z2 × p × q])/d2, where z = 95% confidence interval, p = prevalence of knowledge 16%, q = 1 – p, d = error ≤5% and a 30% non-response rate.

### Study Instrument and Measures

A questionnaire was utilised to measure the level of knowledge and practice of mothers concerning newborn care. The contents of the questionnaire were adopted from similar studies conducted in Nepal [10] and Kenya [11], assessing newborn care knowledge and practice. The questionnaire was composed of three sections. The first section involved the demographics of the study participants. The second section asked mothers about their awareness of the newborn screening programme, normal birth weight, breast feeding, sanitation, and hygiene, as well as their knowledge about vaccinations. The third part asked the participants about their actual practice with their newborn, including breastfeeding behaviour, vaccination status, and utilisation of health services. Additionally, utilisation of alternative treatments provided by a traditional healer, such as cauterization of cutaneous tissues or use of herbal medications, was assessed.

The content of the questionnaire was reviewed by a paediatric specialist to ensure its suitability to a Saudi population. Face validity of the questionnaire was examined on 10 mothers to test the clarity of the questions and the time needed to fill in the questionnaire. Internal consistency was tested using Cronbach’s Alpha to assess the reliability of the questionnaire, providing a reasonable internal reliability of 0.66.

Data was collected via interviews. These were conducted via trained medical students. Interviews were performed to ensure it was possible to collect data from all mothers, including those who were illiterate.

### Data Analysis

Statistical analysis was performed using the Statistical Package for the Social Sciences (IBM Corp, Armonk, NY, USA) version 22. Frequency and proportions were used to summarise categorical and binary variables. Mean and standard deviation were used to summarise continuous variables. A scoring system was developed to indicate mothers with high or low knowledge and mothers with adequate or inadequate practice. The scoring was based on summing the number of correct or adequate responses, where one point was given for each correct answer or adequate practice. To dichotomize level of knowledge and practice, cut-off points were estimated based on visualisation of data and calculating medians. After estimation of cut-off points, participants who scored lower values than the cut-off points were labelled as subjects with low knowledge or inadequate practice and those who scored higher than cut-off points were labelled as subjects with high knowledge or adequate practice. Logistic regression, Chi square test, and Fisher Exact test were used to assess the presence of statistically significant associations between demographic factors and level of knowledge and practice adequacy. A p-value of less than 0.05 was presumed statistically significant for the applied statistical tests.

## Results

### Summary of Study Participants

A total of 450 mothers participated in the current investigation. Table 1 illustrates demographics of the included mothers. Ages ranged between 17 and 46 years, where a majority of mothers were over 32 years old. The mean age of the youngest child varied between 1 month to 48 months, and a majority of youngest children were over 24 months old. A majority of mothers had one child or two at the time of recruitment, had a university degree, were housewives, and had a monthly income between 5000 and 10,000 Saudi Riyals. About 91% of participating mothers declared they utilized antenatal care during their pregnancies, and 99% of the mothers reported having their last child born at a hospital.

**Table 1:** Demographic characteristics of 450 mothers in Jazan region.

Variables*	Frequency	Percent

Age		
<26 years	142	31.8%
Between 26 and 32 years	145	32.5%
>32 years	159	35.7%
Age of the youngest (in months)		
<10	147	32.7%
Between 10 and 24	123	27.3%
>24	180	40%
Parity		
1–2	223	49.6%
3–4	127	28.2%
5 or more	100	22.2%
Level of education		
Illiterate	19	4.2%
Primary school	30	6.7%
Intermediate school	32	7.1%
Secondary school	123	27.3%
University	246	54.7%
Occupation		
Housewife	224	52.1%
Employed	123	28.6%
Student	83	19.3%
Monthly income (Saudi Riyals)		
Less than 5000	154	34.5%
Between 5000 and 10,000	170	38.1%
More than 10,000	122	27.4%
Antenatal care visits		
Yes	408	90.7%
No	42	9.3%

* Missing 4 cases for age, 20 cases for occupation, and 4 cases for monthly income.

### Level of Knowledge

Participating mothers’ knowledge concerning newborn care is summarized in Table 2. The mean level of knowledge was 11.85/16 [SD: 2.6]. A majority of participating mothers were able to give correct answers, where correct answers exceeded 70% for 11 out of 16 survey items. The question concerning importance of feeding a newborn in the first hour of life scored the highest proportion of correct answers. However, questions concerning normal frequency of newborn defecation per day, the need to postpone bathing a newborn for 24 hours after birth, and awareness about neonatal screening programs scored the lowest proportion of correct answers.

**Table 2:** Knowledge of 450 mothers from Jazan, Saudi Arabia, concerning newborn care.

Survey item	Correct answer	

	Frequency	Percentage

Awareness of the neonatal screening program	255	56.7%
Normal birth weight	332	73.8%
Feeding of newborn in the 1st hour of life	406	90.2%
Importance of colostrum	361	80.2%
Breastfeeding interval	393	87.3%
Importance of pre-lactal feeding	308	68.4%
Importance of drying newborn	362	80.4%
Importance of wrapping newborn	362	80.4%
Keeping the newborn close to his mother.	381	84.7%
The need to postpone bathing of newborn for 24 hours after birth	210	46.7%
Hand washing by mothers before feeding baby	368	81.8%
Nipple cleaning before breastfeeding	394	87.6%
Awareness of the Saudi immunization schedule	389	86.4%
Believing that vaccines can be harmful to newborns	397	88.2%
Normal frequency of newborn defection per day	180	40.1%
Knowing that inappropriate feeding positions can cause ear and chest infection	266	59.1%

### Practice Adequacy

Practice of care is described in Table 3. The mean score for practice adequacy was 7.11/10 [SD: 1.5]. Among measured newborn care practices, 93% of responding mothers indicated vaccinations according to a vaccination schedule. The practice scoring the lowest adherence was postponing bathing the newborn for 24 hours after birth. Furthermore, about 33% of mothers reported not initiating breastfeeding within the first hours after birth. Additionally, 27% of mothers declared using alternative treatments, such as herbal medication and cauterization, to treat their newborn instead of seeking professional health care from available health services.

**Table 3:** Practice of 450 mothers from Jazan, Saudi Arabia toward their newborns.

Questions	Appropriate practice response	

	Frequency	Percentage

Initiation of breastfeeding in the 1st hour after birth	303	67.3%
Colostrums feeding	350	77.8%
Feeding interval	359	80.5%
Pre-lactal feeding	380	84%
Newborn wrapped immediately after birth	401	90.3%
Keeping newborn close to mother	356	80%
Postponing bathing newborn for 24 hours after birth	258	57.8%
Up to date vaccination of child	416	93.3%
Did not use alternative treatments to treat child	328	72.9%

### Association with Demographic Factors

The association between demographic characteristics and level of knowledge and practice adequacy is illustrated in Table 4. The strongest association with knowledge level was apparent when comparing education level, employment status, and adherence to antenatal visits during pregnancy. These observed differences were statistically significant (p < 0.05). The odds of having a high level of knowledge was greater among mothers with university or postgraduate education in comparison to mothers with lower education levels. Similarly, the odds of having a high level of knowledge was higher among employed mothers in comparison to unemployed or student mothers.

**Table 4:** Association between demographic factors and level of knowledge and practice of care for 450 mothers from Jazan, Saudi Arabia.

Demographic variables	Total	Odds of high knowledge			Odds of competent practice		

		OR*	[95%CI]	P value	OR*	[95%CI]	P value

**Age**							
<26 years	142	Reference			Reference		
Between 26 and 32	145	1.31	0.81–2.11	0.266	1.50	0.91–2.46	0.105
>32 years	159	1.05	0.66–1.66	0.820	**1.82**	**1.11–2.98**	**0.017**
**Education level**							
Illiterate/primary	49	Reference			Reference		
Intermediate/secondary	155	**2.02**	**1.04–3.89**	**0.036**	2.59	0.78–8.53	0.110
University and above	246	**3.67**	**1.94–6.93**	**0.000**	1.85	0.58–5.85	0.294
**Occupation**							
Housewife	244	Reference			Reference		
Employee	123	**2.97**	**1.82–4.86**	**0.000**	**1.83**	**1.10–3.02**	**0.018**
Student	83	1.54	0.92–2.58	0.095	1.01	0.59–1.71	0.963
**Monthly income (Saudi Riyals)**							
less than 5000	158	Reference			Reference		
5000–10,000	170	1.103	0.70–1.71	0.664	1.52	0.95–2.44	0.079
more than 10,000	122	1.52	0.93–2.50	0.094	1.26	0.76–2.09	0.368
**Antenatal care visits**							
No	42	Reference			Reference		
Yes	408	**2.61**	**1.36–4.99**	**0.004**	1.8	0.94–3.43	0.075
**Age of youngest (months)**							
<10	147	Reference			Reference		
Between 10 and 24	123	0.70	0.43–1.15	0.169	0.89	0.54–1.48	0.675
>24	180	0.75	0.47–1.18	0.215	1.50	0.92–2.42	0.090
**Number of children**							
1–2 children	223	Reference			Reference		
3–4 children	127	1.04	0.66–1.64	0.852	1.44	0.89–2.33	0.135
5 or more	100	0.78	0.48–1.27	0.325	1.42	0.847–2.40	0.182

* Odds ratio.

The association between demographic characteristics and practice adequacy level seems to differ when compared to associations with knowledge. Age appeared to be associated with practice, where mothers older than 32 had greater odds of having a high level of competency (p = 0.017). Education level and attending antenatal care visits did not seem to show a statistically significant association with practice. Finally, employed mothers were found to have higher odds of adequate practice compared to housewives or student mothers (p = 0.018).

Having found that 27% of mothers utilized alternative treatments, such as cauterization and herbal medications, as a health care option for their newborn could be worth investigating. Demographic factors associated with choosing to use alternative treatments were tested. Education level appeared to be a factor: 56% of illiterate mothers and 40% of mothers with primary education sought cauterization and alternative medicine for their newborn (p = 0.009). Additionally, 50% of mothers whom did not attend antenatal care visits during their pregnancy reported using alternative treatments for their newborn (p = 0.0003).

About 60% of mothers who did not attend antenatal care visits scored a lower level of knowledge. Nonetheless, given the retrospective nature of this investigation, it is not clear whether a higher level of knowledge would increase adherence toward antenatal care visits or whether knowledge of newborn care was enhanced due to attending antenatal visits.

The univariate logistic regression revealed several associations between demographic variables and level of knowledge and practice adequacy. However, occupation and tendency to attend antenatal care visits were found to be related to education level, which interfered with assuming these variables as independent variables in a multivariate regression. However, examining the associations between level of education, occupation, and attendance of antenatal care was performed using Fisher Exact test (Table 5). It can be observed that education level has a statistically significant association with both tendency of attending antenatal care visits and occupation. These associations may indicate that odds of having a higher level of knowledge among employed mothers and mothers attending antenatal care visits are partially explained by their higher education levels.

**Table 5:** Association between education level, attendance of antenatal care visits, and occupation in a sample of 450 mothers from Jazan, Saudi Arabia.

Education level	Antenatal care visits			Occupation			

	Yes	No	P value	Housewife	Employee	Student	P value

Illiterate/primary	35 (71.4%)	14 (28.6%)	<0.001*	48 (98%)	0 (0%)	1 (2%)	<0.001*
Intermediate/secondary	139 (89.6%)	16 (10.4%)		126 (81.3%)	11 (7.1%)	18 (11.6%)	
University and above	229 (93%)	11 (7%)		70 (28.5%)	112 (45.5%)	64 (26%)	

* Fisher Exact test.

## Discussion

This study is a cross-sectional study assessing the level of knowledge and adequacy of practice concerning newborn care among mothers in different areas of Jazan, Saudi Arabia. The majority of mothers were not aware of the normal defecation frequency of newborns, the importance of postponing newborn bathing for 24 hours after birth, and the newborn screening programme. The knowledge of mothers concerning newborn care appeared to be associated with education level, employment status, and attendance of antenatal care visits during pregnancy. More than 90% of mothers reported up to date vaccination of their children and reported immediately wrapping their children after birth. However, 42% of mothers reported bathing their newborns during the first 24 hours after birth. The age of mothers and their employment status appeared to be associated with adequacy of practice, indicating better practice among older and employed mothers.

As far as we know, studies assessing the level of knowledge and practice of mothers concerning newborn care in Saudi Arabia are lacking. However, several studies were found investigating a particular area within newborn care, such as breastfeeding, in several cities in Saudi Arabia. The proportion of mothers who initiated breastfeeding within the first hour of birth was found to be 31% in Abha [12], 77% in Al-Hassa [13], and 24% in Al-Qasim [14].

The proportion of mothers who initiated breastfeeding within the first hour after birth was 67% in our study, which is similar to the findings of the Al-Hassa study, but much higher than the findings in the Abha and Al-Qasim studies. A possible explanation for this marked variation is related to sampling; unlike our sample, which recruited a wide spectrum of mothers with a wide distribution of demographic variables, the Abha and Al-Qasim studies were limited to school teachers and not the general community.

A study by Al-Ayed assessed the level of knowledge of mothers concerning child health matters, including topics related to children older than one year [9]. In the Al-Ayed study, an overall satisfactory level of knowledge of mothers was reported, which is similar to our findings, where the majority of mothers were able to give correct answers to survey items.

Within our sample, 42% reported a lack of awareness of newborn screening programmes. This notion is supported by the findings of the study by Al-Sulaiman et al. which assessed the knowledge and attitude of Saudi mothers towards newborn screening. Their investigation reported an overall positive attitude but with limited awareness about the screening tests [15].

Several international studies have assessed the knowledge and practice of mothers concerning newborn care in South Asian and African countries, such as India [16], Ethiopia [17], Nepal [10], Kenya [11], Pakistan [18], Sri Lanka [19], and Bangladesh [20]. It can be observed that a majority of studies assessing knowledge and practice of mothers concerning newborn care are mainly performed in underdeveloped or developing countries. This can be explained by the fact that the highest rates of neonatal mortalities are reported in South Asian, African, and Latin American countries [21]. However, variations in the magnitude of knowledge and the practice of mothers between these societies are expected given the variations in sociodemographic factors and the quality of healthcare services. For example, the proportion of home deliveries in the study conducted in Pakistan was 18%, while only 1% reported giving birth to their last child at home in our sample.

The associations between demographic variables and the levels of knowledge and practice of care assessed in international studies is similar to the findings of our investigation. For example, a study conducted in Sri Lanka [19], including a sample of 446 mothers, reported lower levels of knowledge among unemployed mothers and mothers with delayed antenatal care visits. Nonetheless, a large scale study conducted in Bangladesh [20], including a sample of 6150 mothers, reported that mothers with higher education levels were likely to report adequate newborn care practice, which was not observed in our sample.

A study by Amolo et al. assessed mothers’ levels of knowledge according to attending antenatal care and receiving newborn care education during pregnancy in Kenya [22]. The study found that mothers who attended antenatal care visits and received education concerning newborn care during pregnancy scored higher levels of knowledge. Furthermore, in an international investigation including 57,643 infants/mothers from Argentina, Democratic Republic of Congo, Guatemala, India, Pakistan, and Zambia, it was observed that maternal level of education impacted knowledge regarding newborn care, where training mothers with a lower education was associated with reduction of stillbirth rates [23]. These finding are similar to our finding concerning the association between education level and knowledge regarding newborn care.

This study had multiple areas of strength and weakness. This study was able to assess the gaps in knowledge and inadequacy in practice of mothers in Jazan concerning newborn care. Sampling mothers with different demographic backgrounds enabled assessment of the associations between demographics and knowledge and practice levels. Although excluding mothers with children older than 5 years was done to reduce practice recall bias, we cannot ignore the possibility of its occurrence given the subjective nature of the assessment tool. Finally, limiting this study to mothers in Jazan region hinders the ability to generalise the findings to other regions of Saudi Arabia.

Finding several gaps in the knowledge and adequacy of practice of mothers in Jazan region concerning newborn care mandates the development of strategies and the commencement of initiatives to enhance the quality of newborn care. Attending antenatal care visits during pregnancy appears to be associated with the level of knowledge and the adequacy of practice concerning newborn care. However, 42 mothers from our sample reported not attending antenatal care visits during their pregnancies. The factors influencing attendance is an area for further research. Additionally, 122 mothers reported using herbal medications or cauterisation for their newborns; a majority of these mothers reported lower levels of education. It is worth investigating why these mothers did not favour seeking conventional health services and the possible harm caused to the well-being of the newborn.

## Conclusions

Proportions of correct answers measuring knowledge and practice adequacy varied between 40% to 93%, indicating variability of mothers’ knowledge and practice concerning newborn care in Jazan region. Knowledge and practice appeared to be associated with demographic factors, such as levels of education, age, and attending antenatal care visits. Further assessment is needed to examine the factors associated with attendance, antenatal care visits during pregnancy, and the factors associated with mothers’ choices to opt for alternative treatments for their newborns.
